# Evaluation of centre‐specific machine learning models in predicting 2‐year outcomes of hip arthroscopy for mixed femoracetabular impingement syndrome

**DOI:** 10.1002/jeo2.70477

**Published:** 2025-10-31

**Authors:** Gang Yang, Jiali Kang, Fan Hu, Yin Pei, Dingge Liu, Zhihua Zhang, Kaiping Liu, Langran Wang, Xi Gong, Haijun Wang, Shuangshuang Deng, Ruijie Liu, Xin Zhang

**Affiliations:** ^1^ Beijing Key Laboratory of Sports Injuries, Department of Sports Medicine, Peking University Third Hospital Institute of Sports Medicine of Peking University Beijing Haidian China; ^2^ Department of Epidemiology and Biostatistics, School of Public Health Peking University Health Science Centre Beijing China; ^3^ Tianjin University of Sport Tianjin China

**Keywords:** femoroacetabular impingement syndrome, hip arthroscopy, machine learning prediction models, patient‐reported outcomes scores, preoperative factors

## Abstract

**Purpose:**

To construct a centre‐specific machine learning (ML) prediction model based on preoperative factors. It was hypothesised that the ML prediction model would accurately predict whether patient‐reported outcome scores (PROs) over at least 2 years would reach the minimal clinically important difference (MCID).

**Methods:**

A retrospective analysis was performed on mixed‐type femoroacetabular impingement syndrome (FAIS) patients who had hip arthroscopy at our institution between 2016 and 2018. The primary outcome was the rate of achieving MCID in PROs assessed at least 2 years after surgery, PROs included the hip outcome score‐activities of daily living (HOS‐ADL), modified Harris Hip Score (mHHS), visual analogue scale (VAS) for pain and international hip outcome tool‐12 (iHOT‐12), assessed at a minimum of 2 years postoperatively. Preoperative patient features were selected using the least absolute shrinkage and selection operator (LASSO) algorithm. Three ML models were constructed using balanced sample data and optimal feature subsets: logistic regression (LR), support vector machine (SVM) and random forest (RF). Model performance was assessed using the area under the receiver operating characteristic curve (AUROC) and the concordance index (C‐index). Model interpretations were conducted using the SHapley Additive explanation (SHAP) method.

**Results:**

A total of 210 patients (48.1% female) were included. The LR, SVM, RF models had AUROC 0.76 (0.61–0.83), 0.89 (0.80–0.94), 0.99 (0.98–1.00), respectively, and C‐index 0.74 (0.65–0.82), 0.86 (0.81–0.90), 0.95 (0.93–0.96), respectively. Preoperative symptom duration, preoperative HOS‐ADL, hip joint space and preoperative alpha angle were identified as the most important predictors.

**Conclusion:**

Among the three ML prediction models, RF performed best in predicting whether PROs reached MCID, demonstrating excellent discriminative ability, calibration and robustness. This indicates that individualised and robust ML prediction models for outcome prediction based on preoperative factors are feasible even with limited amounts of centre‐specific data.

**Level of Evidence:**

Level III.

**Abbreviations**
3D: three‐dimensional
AL: anterolateral
AP: anteroposterior
AUROC: area under the receiver operating characteristic curve
C‐index: concordance index
CT: computed tomography
FAIS: femoroacetabular impingement syndrome
FNR: false negative rate
HOS‐ADL: hip outcome score‐activities of daily living
iHOT‐12: international hip outcome tool‐12
LASSO: least absolute shrinkage and selection operator
LCEA: lateral central‐edge angle
LR: logistic regression
MAP: midanterior portal
MCID: minimal clinically important differences
mHHS: modified Harris Hip Score
ML: machine learning
MRI: magnetic resonance imaging
OA: osteoarthritis
PROs: patient‐reported outcomes scores
RF: random forest
SHAP: SHapley Additive explanation
SMOTE: synthetic minority oversampling technique
SVM: support vector machine
VAS: visual analogue scale

## INTRODUCTION

Femoroacetabular impingement syndrome (FAIS) is the leading cause of hip pain in young adults. In addition, FAIS is also a significant contributor to hip osteoarthritis (OA) [7, 37, 42]. FAIS is typically categorised into cam type, pincer type or mixed type [2, 7, 47]. Labral and cartilage injuries in the hip can be treated with arthroscopic surgery, and for normalising the anatomical morphology of the hip joint in mixed FAIS, intraoperative cam resection and pincer resection are essential [12, 13].

Minimal clinically important difference (MCID) is an important marker of surgical success, it is the minimal beneficial change that can be perceived by patients, and patient‐reported outcome score (PROS) is a widely used outcome tool [25, 32, 40, 44]. For surgeons, one of their key goals is to improve the MCID attainment rate of postoperative patients by various means. In addition to efforts to improve the surgical technique of surgeons, measures such as optimising patient screening and prehabilitation can be used to improve MCID attainment rate [24, 36]. Prediction models for PROs can improve shared decision‐making and patient counselling [14]. Meanwhile, machine learning (ML) techniques have attracted interest in the field of predictive analytics in medicine. Currently, the ML prediction model has been recognised as capable of predicting patients' postoperative PROs, thereby improving and optimising the quality of medical services [9]. Various ML techniques and simple neural networks provide higher predictive power compared to more traditional modelling techniques [39]. The preoperative ability to predict which patients with FAIS will achieve the MCID after hip arthroscopy helps determine the appropriateness and timing of surgery, so as to help doctors and patients choose more appropriate treatment time and method [9].

Prediction models based on multicentre clinical data usually have the characteristics of large sample size and strong versatility, which can provide a reference for all surgeons. However, patient characteristics and surgical techniques vary among centres. Thus, surgeons can train and apply centre‐specific prediction models to more accurately predict outcomes after hip arthroscopy for better clinical decision making. The aim of this study was to develop centre‐specific ML prediction models based on preoperative factors, to predict at least 2‐year postoperative outcomes for patients with mixed‐type FAIS after hip arthroscopy. It was hypothesised that a centre‐specific ML prediction model would accurately predict whether PROs over at least 2 years would reach MCID.

## MATERIALS AND METHODS

### Patient selection

Approval for the study was granted through the institutional review board (IRB) of Peking University Third Hospital (Number: M2019193). Patients who underwent primary hip arthroscopic surgery for FAIS at our institution between August 2016 and December 2018 were retrospectively analysed. All arthroscopic surgeries were performed by two senior authors (X.Z. and J‐Q.W.). Each of them performs more than 50 hip arthroscopic procedures annually.

**Figure 1:**
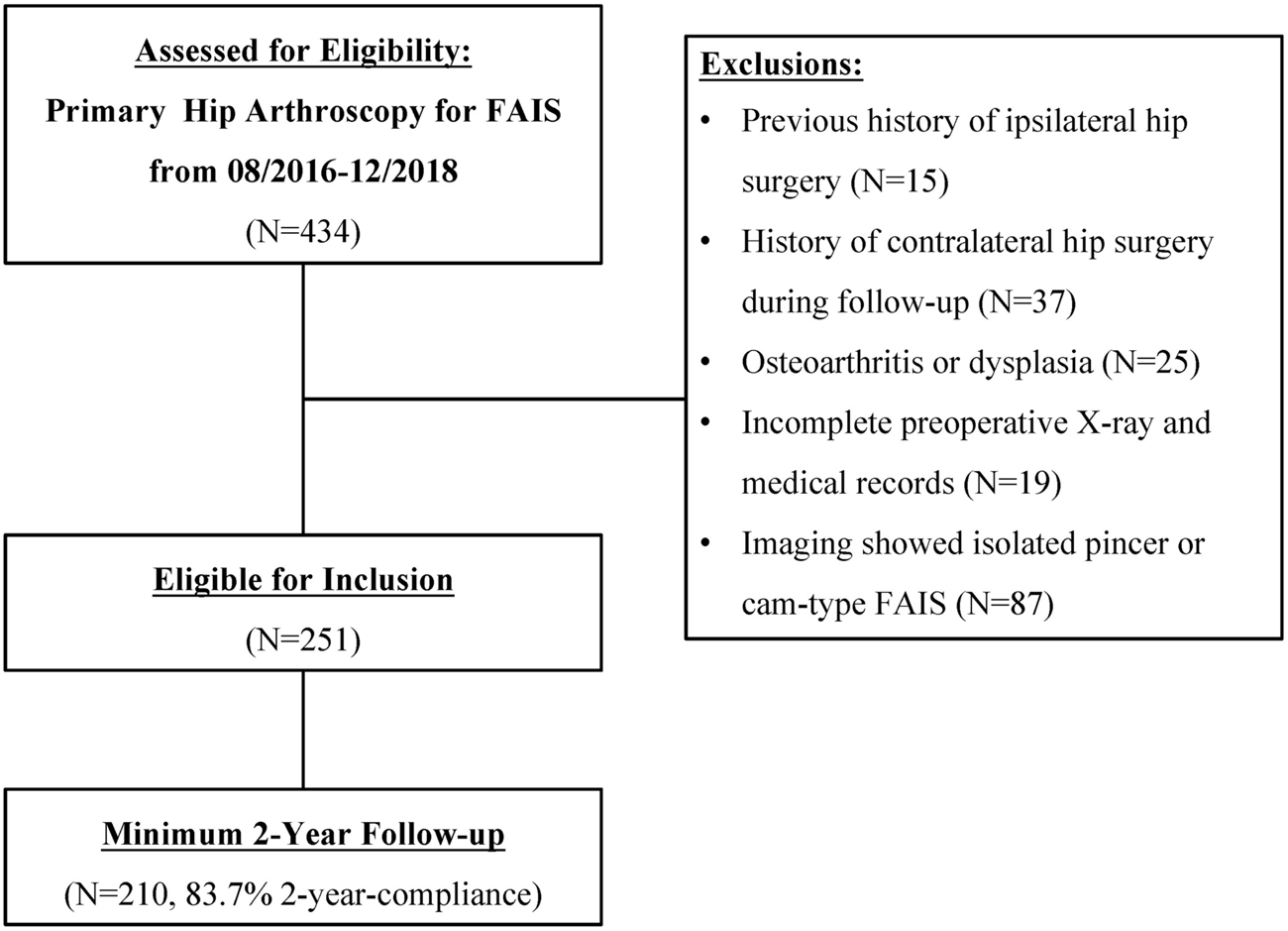
Patient selection. A total of 210 patients who met the inclusion criteria and complete data at least 2 years of follow‐up were included in the study. FAIS, femoroacetabular impingement syndrome.

Inclusion criteria were as follows: (1) patients underwent primary hip arthroscopy due to FAIS; (2) patients were followed up for at least 2 years. Exclusion criteria were as follows: (1) Previous history of ipsilateral hip surgery. (2) History of contralateral hip surgery during follow‐up. (3) Preoperative lateral central‐edge angle (LCEA) < 25°, moderate to advanced OA (Tönnis grade ≥2). (4) Incomplete preoperative X‐ray and medical records. Arthroscopic surgery was performed in 434 hips. After exclusions, a total of 251 hips were deemed eligible. Ultimately, 210 hips were followed for at least 2 years and included in the study (Figure 1), either by telephone or in response to questions on the scale.

### Radiographic examination

All patients underwent anteroposterior (AP) pelvic and 45° Dunn lateral radiography, unilateral hip computed tomography (CT) and magnetic resonance imaging (MRI). A picture archiving and communication system (PACS; GE Healthcare) was used; The images were independently evaluated by an experienced radiologist, and all measurements were confirmed by a senior author (X.Z.). The LCEA and alpha angle were measured on AP pelvic radiographs. While the alpha angle was measured on 45° Dunn lateral radiographs. Cam deformity was defined as an alpha angle >55° [26]. Additionally, the LCEA was measured on AP pelvic radiographs, with pincer deformity defined as an LCEA ≥ 40° or a crossing sign [47]. Labral and articular cartilage were evaluate using 3.0‐T MRI. Cam deformity was assessed before and after operation using three‐dimensional (3D) CT.

### Surgical technique

The patient was positioned in a modified supine position on a standard hip traction table, with perineum protection during limb traction. Labral tears have been treated with debridement, repair, or reconstruction, depending on the extent of the injury: Labral tears were repaired using suture anchor fixation (Smith & Nephew) when possible. In cases of irreparable labral tears, autograft gracilis transplantation could be considered for labral reconstruction. Acetabuloplasty was performed using a motorised bur, to create a bleeding bone bed for labral healing. Chondroplasty was performed for partial‐thickness cartilage lesions and chondral flaps, using an arthroscopic shaver and/or electrocautery device (Smith & Nephew). Subspine impingement was diagnosed based on preoperative imaging and confirmed with a preoperative subspine blocking test guided by ultrasound. Resection of the subspine deformity back to the level of the acetabular sourcil was typically performed. After addressing central compartment pathologies, the arthroscope was introduced into the peripheral compartment to decompress cam deformities using a high‐speed bur (Smith & Nephew). Adequate resection of the cam deformity was confirmed through dynamic examination and fluoroscopic imaging. Finally, the joint capsule of all patients was routinely full closure at the end of the procedure using nonabsorbable Orthocord (DePuy Mitek).

### Postoperative rehabilitation

With well supervision from our physical therapy team, all patients followed a standardised prescribed rehabilitation protocol [46]. The rehabilitation was divided into four phases. The first phase of the rehabilitation protocol emphasised isometric contractions and passive range of motion exercises. The second phase focused on establishing a regular gait pattern and restoring full range of motion. The emphasis of the third phase shifted towards rebuilding lower extremity strength and normal functional activities. The final phase of rehabilitation centred on reintegrating preinjury higher‐level activities.

### Assessment of PROs

PROs included scores for the hip outcome score‐activities of daily living (HOS‐ADL), modified Harris Hip Score (mHHS), visual analogue scale (VAS) for pain, international hip outcome tool‐12 (iHOT‐12) and hip outcome score‐sports subscale (HOS‐SS) [25, 44]. However, in this study, half of the patients did not exercise regularly, so HOS‐SS was not calculated. Patients completed surveys at the hospital before surgery and via a telephone interview after surgery. All assessments were performed in the patient's native language. Additionally, MCID and patient acceptable symptom state (PASS) were calculated to identify meaningful outcome improvements [17]. The MCID values used in this study were determined based on previous research: [1, 22, 33] 1.5 points for VAS pain, 8.7 points for mHHS, 8.3 points for HOS‐ADL, and 13.0 points for iHOT‐12. The PASS cutoffs were 1.91 for VAS pain, 83.3 for mHHS, 88.2 for HOS‐ADL, and 72.2 for iHOT‐12.

### Development of ML prediction models

Initially, descriptive statistics assessed the overall sample characteristics. Next, feature screening utilised the Least Absolute Shrinkage and Selection Operator (LASSO) [45]. Using the balanced data from the optimal feature subset, three ML models were constructed—logistic regression (LR) [28], support vector machine (SVM) [6] and random forest (RF) [3], to predict whether the four PROs reach the MCID. And model interpretation and knowledge mining were performed based on the SHapley Additive exPlanations (SHAP) method [19]. SPSS (version 24.0) facilitated the initial data description, while Python (version 3.8.10) supported all subsequent analyses. The code and supporting documents used to generate the model can be accessed at https://github.com/ailiwood/ML-FAIS-MCID/tree/main.

### Statistical analysis

The primary outcome of this study was the performance of the prediction model, and the secondary outcomes were the preoperative factors affecting the postoperative outcomes of patients. Continuous variables fitting a normal distribution, such as age and BMI, were presented using mean ± standard deviation. Those not fitting a normal distribution were presented using median (interquartile range). Categorical variables, such as gender, were shown using frequency (percentage). Statistical comparisons of PROs, LCEA and alpha angles before and after surgery utilised a two‐tailed paired Student t‐test. A *p*‐value below 0.05 was deemed statistically significant.

To address the common issue of overfitting in ML and align with prior studies [15], the LASSO model was used for feature selection. This approach helps remove irrelevant or redundant features, reducing modelling time and potential noise, thereby enhancing model accuracy [38]. The lasso was designed to predict changes in PROs scores pre‐ and postoperatively (change values were the basis for determining MCID). Parameters were optimised using a grid search algorithm to develop an optimal model for each PROs score, selecting the feature subset for the predictive model.

Since imbalance reduces the predictive performance and generalisation ability of the model, sample balancing is performed before formal modelling. The ratio of positive samples in the raw data is more than 9:1, and downsampling would result in a significant loss of information. Therefore, the synthetic minority oversampling technique (SMOTE) is used [27]. Then ML models were developed.

In the model training and evaluation phases, GridSearchCV was used to identify optimal hyperparameter combinations [35]. And a 10‐fold cross‐validation method for model evaluation.

Due to the highly imbalanced nature of the original dataset, accuracy alone does not fully capture the model's precision. Consequently, the F1 score and the false negative rate (FNR) were also reported. For discrimination, the area under the receiver operating characteristic curve (AUROC) and the concordance index (C‐index) were evaluated. While AUC assesses the model's overall ability to distinguish between positive and negative cases, the C‐index more specifically evaluates the relative risk ranking among samples. Additionally, this study includes the Brier score to gauge and compare the calibration of clinical prediction models. The Brier score measures the mean square deviation between the predicted probability and the actual outcome; thus, a lower Brier score indicates a better predictive ability of the model.

In the phases of model interpretation and knowledge mining, SHAP was used. A key advantage of SHAP values is their consistency, which ensures the reliability of the feature importance assessment. The robustness and transparency of Shap have facilitated its widespread adoption in various research areas [10, 18].

## RESULTS

### Patient characteristics

A total of 210 patients (51.90% male vs. 48.10% female) were included in the final analysis. The patients' basic information, physical examination findings and hip pain history. Statistically significant differences were observed between preoperative and postoperative alpha angles (65.85 ± 8.15 vs. 44.11 ± 5.11; *p* < 0.001) and between preoperative and postoperative LCEA (33.26 ± 6.46 vs. 31.17 ± 5.52; *p* < 0.001). Throughout the follow‐up period, there was a significant increase in mHHS (62.51 ± 7.22 vs. 89.09 ± 8.97, *p* < 0.001), HOS‐ADL (64.01 ± 8.21 vs. 89.27 ± 8.67; *p* < 0.001), iHOT‐12 (41.02 ± 6.79 vs. 73.39 ± 10.33, *p* < 0.001) and a significant decrease in VAS pain scores (6.00 ± 1.37 vs. 1.72 ± 1.45; *p* < 0.001) (Table 1).

**Table 1:** Characteristics of the study cohort (*N* = 210 hips).

Characteristics	Mean ± SD or *n* (%)
Age, years	37.58 (±9.64)
Sex	
Male	109 (51.90%)
Female	101 (48.10%)
Height, cm	169.59 (±7.96)
Weight, kg	67.83 (±12.49)
BMI	23.45 (±3.17)
Symptom duration, months	17.67 (±15.43)
Tönnis grade	
0	130 (61.90%)
1	80 (38.10%)
Joint space, mm	4.53 (±0.80)
Diameter of femoral head, mm	52.51 (±5.60)
Preoperative symptom duration >2 year	29(13.81%)
Follow‐up time, months	38.68 (±7.94)
Alpha angle, deg	
Preoperative	65.85 ± 8.15
Postoperative	44.11 ± 5.11
LCEA, deg	
Preoperative	33.26 ± 6.46
Postoperative	31.17 ± 5.52
mHHS	
Preoperative	62.51 ± 7.22
Postoperative	89.09 ± 8.97
Achievement of PASS	163 (77.61%)
Achievement of MCID	199 (94.76%)
HOS‐ADL	
Preoperative	64.01 ± 8.21
Postoperative	89.27 ± 8.67
Achievement of PASS	141 (67.14%)
Achievement of MCID	198 (94.28%)
iHOT‐12	
Preoperative	41.02 ± 6.79
Postoperative	73.39 ± 10.33
Achievement of PASS	121 (57.62%)
Achievement of MCID	197 (93.80%)
VAS	
Preoperative	6.00 ± 1.37
Postoperative	1.72 ± 1.45
Achievement of PASS	113 (53.81%)
Achievement of MCID	195 (92.86%)

Abbreviations: BMI, body mass index; HOS‐ADL, hip outcome score activities of daily living; iHOT12, international hip outcome tool‐12; LCEA, lateral centre‐edge angle; MCID, minimal clinically important difference; mHHS, modified Harris Hip Score; PASS, patient acceptable symptom state; VAS, visual analogue scale.

### Variable screening

Table 2 presents the results of variable selection performed using LASSO for each of the four patient‐reported outcomes. A check mark (‘√’) indicates that the variable was retained in the optimal subset for that outcome, signifying its potential importance in predicting whether the MCID would be achieved. These selected feature subsets served as the inputs for subsequent ML model training and performance comparison. Variables in bold were consistently selected across all four outcomes, highlighting predictors of broad clinical relevance.

**Table 2:** Results of variable screening for LASSO.

Characteristics	mHHS	HOS‐ADL	iHOT‐12	VAS
**Age, years**	√	√	√	√
Sex		√		
Height, cm		√	√	
Weight, kg		√		√
BMI				
**Diameter of femoral head, mm**	√	√	√	√
Joint Space, mm				√
Tonnis grade				
**Symptom duration, months**	√	√	√	√
Preoperative symptom duration >2 year				
**Preoperative alpha angle, deg**	√	√	√	√
Preoperative LCEA, deg		√		√
**Preoperative mHHS**	√	√	√	√
Preoperative HOS‐ADL		√	√	√
Preoperative iHOT‐12			√	√

*Note*: Bold characteristics, indicating significance for all PROs.

Abbreviations: BMI, body mass index; HOS‐ADL, hip outcome score activities of daily living; iHOT12, international hip outcome tool‐12; LASSO, least absolute shrinkage and selection operator; LCEA, lateral centre‐edge angle; mHHS, modified Harris Hip Score; VAS, visual analogue scale.

### Model comparison

**Figure 2:**
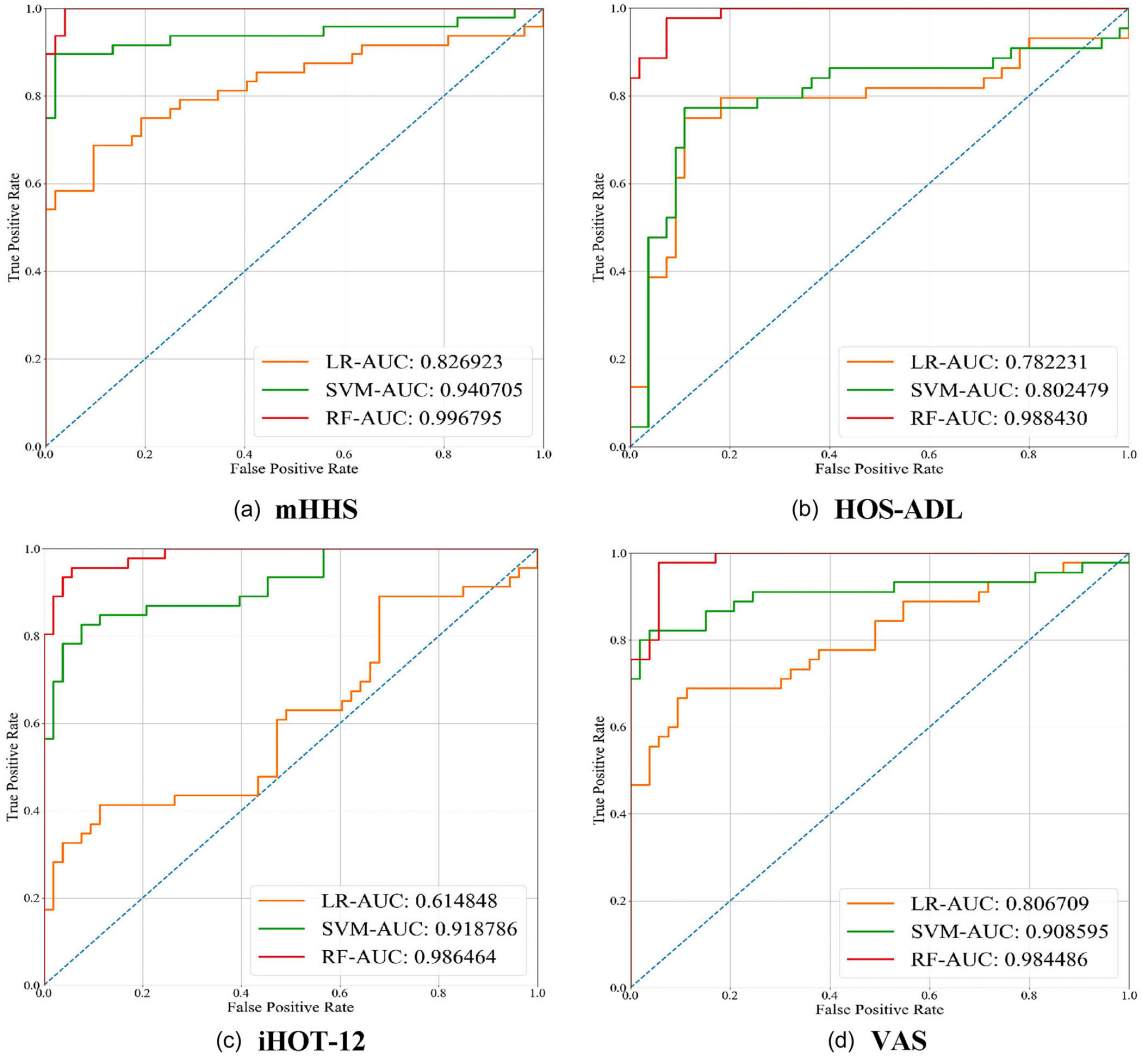
The ROC curves for the three models on the four datasets and reports the AUC. (a–d) The results on the four datasets mHHS, HOS‐ADL, iHOT‐12 and VAS, respectively. AUC, area under the ROC curve; HOS‐ADL, hip outcome score‐activities of daily living; iHOT‐12, international hip outcome tool‐12; LR, logistic regression; mHHS, modified Harris Hip Score; RF, random forest; SVM, support vector machine; VAS, visual analogue scale.

The RF model outperformed the LR and SVM on all metrics, consistently achieving the highest C index (mean 0.94555) and AUC (mean 0.989043) (Table 3 and Figure 2). In contrast, the LR model exhibited the poorest results, with the highest FNR (mean 0.226875) and a missed diagnosis rate 4.51 times higher than that of SVM and 8.19 times higher than that of RF. The RF model also attained the lowest Brier score, indicating superior model calibration. Further analysis of standard deviation in model evaluation metrics confirms the robustness of the RF model, which consistently exhibits the lowest variability across datasets. Considering its superior discriminatory power and stability, the RF model is recommended for predicting MCID in the clinical setting.

**Table 3:** Performance comparison of the three models on the four datasets.

Data		mHHS		HOS‐ADL		iHOT‐12		VAS	
Model	Metric	Mean	STD	Mean	STD	Mean	STD	Mean	STD
LR	F1 score	0.73	0.07	0.81	0.04	0.65	0.10	0.76	0.05
	FNR	0.28	0.09	0.12	0.08	0.31	0.11	0.20	0.10
	C‐index	0.74	0.07	0.82	0.04	0.65	0.10	0.76	0.04
	Brier score	0.26	0.07	0.18	0.04	0.35	0.10	0.24	0.05
SVM	F1 score	0.89	0.04	0.80	0.04	0.84	0.07	0.89	0.04
	FNR	0.01	0.03	0.10	0.06	0.05	0.04	0.04	0.05
	C‐index	0.90	0.04	0.81	0.05	0.85	0.07	0.89	0.03
	Brier score	0.10	0.04	0.19	0.04	0.15	0.06	0.11	0.04
RF	F1 score	0.95	0.03	0.93	0.03	0.96	0.03	0.93	0.03
	FNR	0.02	0.02	0.04	0.06	0.02	0.04	0.03	0.04
	C‐index	0.95	0.03	0.93	0.03	0.96	0.03	0.93	0.02
	Brier score	0.05	0.03	0.07	0.03	0.04	0.03	0.07	0.03

Abbreviations: C‐index, concordance index; FNR, false negative rate; HOS‐ADL, hip outcome score activities of daily living; iHOT12, international hip outcome tool‐12; LR, logistic regression; MCID, minimal clinically important difference; mHHS, modified Harris Hip Score; RF, random forest; SVM, support vector machine; VAS, visual analogue scale.

### Model interpretation and preoperative predictors analysis

**Figure 3:**
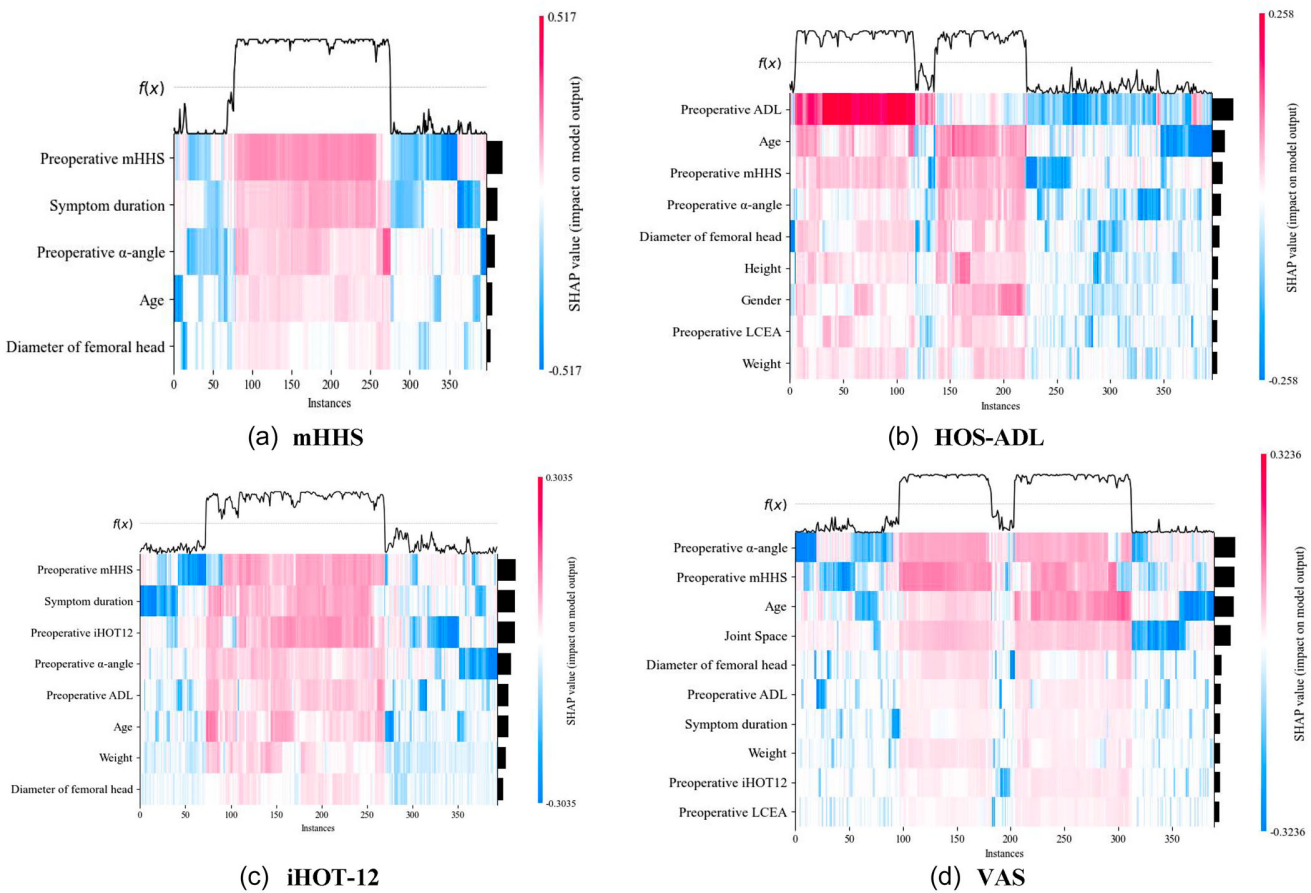
SHapley Additive explanation (SHAP) heat map. Predictors are ordered by importance (top‐to‐bottom). Each thin vertical stripe represents one patient; its horizontal position is that patient's SHAP value. Stripes right of zero (red) raise, and left of zero (blue) lower, the probability of achieving minimal clinically important differences (MCID); darker shades indicate stronger influence. HOS‐ADL, hip outcome score‐activities of daily living; iHOT‐12, international hip outcome tool‐12; mHHS, modified Harris Hip Score; VAS, visual analogue scale.

The results of ranking the importance of features in the random forest model using SHAP, displaying the top five most important scores (Table 4). The SHAP Heat map, with each line representing a feature and the horizontally extended colour bar indicating the feature's impact on a sample (Figure 3). Factors on the left vertical axis are arranged from top to bottom according to predicted strength; each narrow vertical strip (one pixel wide) represents one patient. The strip's horizontal position is that patient's SHAP value for the predictor. Colour encodes the same sign—red for positive, blue for negative—while colour intensity is proportional to the magnitude of influence. Taking ‘age’ as an example, red pixels on the right indicate that, for those older patients, age increases the likelihood of MCID, whereas blue pixels on the left show the opposite effect for younger patients.

**Table 4:** Importance ranking of features based on SHAP.

Targets	Feature	SHAP importance
mHHS‐MCID	Preoperative mHHS	0.18
	Symptom duration	0.13
	Preoperative alpha angle	0.09
	Age	0.07
	Diameter of femoral head	0.05
HOS‐ADL‐MCID	Preoperative HOS‐ADL	0.14
	Age	0.09
	Preoperative mHHS	0.07
	Preoperative alpha angle	0.06
	Diameter of femoral head	0.06
iHOT12‐MCID	Preoperative mHHS	0.08
	Symptom duration	0.08
	Preoperative iHOT12	0.08
	Preoperative alpha angle	0.06
	Preoperative HOS‐ADL	0.05
VAS‐MCID	Preoperative alpha angle	0.10
	Preoperative mHHS	0.09
	Age	0.09
	Joint space	0.08
	Diameter of femoral head	0.04

Abbreviations: HOS‐ADL, hip outcome score activities of daily living; iHOT12, international hip outcome tool‐12; MCID, minimal clinically important difference; mHHS, modified Harris Hip Score; SHAP, SHapley Additive exPlanations; VAS, visual analogue scale.

## DISCUSSION

The most important finding of this study is that by applying ML methods, accurate personalised prediction models can be derived even from small single‐centre cohorts. Three ML prediction models were developed based on patient preoperative factors to predict the outcome of mixed‐type FAIS patients at least 2 years after hip arthroscopy. Among them, the RF ML model has the best performance, with an AUROC of 0.99 and a C‐index of 0.95. And preoperative mHHS, preoperative HOS‐ADL, preoperative mHHS and preoperative alpha angle were identified as the most influential factors affecting the attainment of MCID for mHHS, HOS‐ADL, iHOT12 and VAS scores, respectively.

The main clinical significance of this study is that it provides a strategy for the development and application of FAIS personalised prediction tools. By developing a centre‐specific ML prediction model, surgeons can accurately and reliably inform patients about the possibility of symptom improvement before the surgery. This not only helps patients make inquiries and participate in decision‐making, but also contributes to improving the cost‐effectiveness of FAIS treatment. Different from the traditional way that surgeons tell patients the general success rate of treatment based on traditional experience, this model can predict the postoperative outcome of patients individually according to the previous data of the centre. By applying this model, it is expected to reduce the proportion of patients undergoing unnecessary surgery. Based on individualised predictive analysis, it may even help hospitals better understand their own cost‐effectiveness and find ways to avoid unnecessary surgeries.

Compared with multicentre and large‐sample studies, the prediction model in this study significantly improves the prediction power and interpretability [21, 34]. Pettit et al. [34] developed and used a ML model for predicting MCID attainment in iHOT‐12 after hip arthroscopy based on 1917 patients from the UK's Non‐Arthroplasty Hip Registry. Despite the high quality of the study design and implementation, the AUROC showed that the accuracy of the five prediction models in predicting MCID at 6 months after surgery was fair or poor. The main reason for this is that less than 37% of FAIS patients in this sample data who underwent arthroscopy completed the defined primary outcome, limiting the data set for model development, and of these 37%, many had missing data on baseline patient variables. Martin et al. [21] reported similar findings, they used 5581 patients from the Danish Hip Arthroscopy Registry to predict progression to revision arthroscopy after primary hip arthroscopy, and they concluded that their models had limited clinical utility due to wide discrimination confidence intervals. Perhaps the preferred approach would be to develop a predictive model with data from tens of thousands of patients that could potentially be broadly applicable and also have good accuracy in external data [8]. However, it often requires complex, expensive data collection and sacrifices individualised accuracy. Surgeons in different centres are exposed to different patient characteristics and use different diagnostic criteria, imaging measurements, surgical techniques and postoperative rehabilitation techniques. Therefore, it is still recommended that each centre develop personalised prediction models based on its own clinical data.

Among the prediction models used in this study, LR and SVM are common models used for binary classification tasks in disease prognosis prediction. Introducing the RF model enriches predictive models and confirms its effectiveness, advancing FAIS prognosis prediction. This study pioneers the use of the SHAP method in FAIS prognostic research. The results highlight the importance of factors like patient age and imaging characteristics in surgical intervention effectiveness. SHAP enhances model interpretability, aiding clinical decision‐making. This study significantly improves both predictive capability and interpretability, contributing to FAIS prognostic research.

Among the preoperative patient characteristics, age, femoral head diameter, duration of symptoms, preoperative alpha angle and preoperative mHHS were found to influence the attainment of MCID for all PROs. Specifically, preoperative mHHS, preoperative HOS‐ADL, preoperative mHHS and preoperative alpha angle emerged as the most influential factors determining whether mHHS, HOS‐ADL, iHOT12 and VAS scores reached MCID, respectively. Previous studies have consistently demonstrated that younger age and shorter symptom duration are significantly associated with improved PROs [17, 20, 23, 25]. Therefore, patients should pay attention to their symptoms and do not delay in seeking medical treatment. An increased alpha angle has generally been linked to poorer clinical outcomes in previous research [16, 41]. In our study, both alpha angle and femoral head diameter emerged as important predictors of PROs, indicating that femur‐related factors exert a greater influence on MCID in imaging. The mHHS demonstrates a high predictive power for each PRO, underscoring its reliability as a hip evaluation tool applicable in clinical practice.

In this study, patients exhibited significant reductions in alpha angle (65.85 vs. 44.11, *p* < 0.001) and LCEA (33.26 vs. 31.17, *p* < 0.001) postoperatively. Prior studies have indicated that after cam resection, impingement typically ceases at an alpha angle of 42°–43° [5, 30], consistent with the postoperative alpha angle observed in our study. The normal range of LCEA is generally considered to be 25°–35° [4, 29], consistent with our findings. Previous follow‐up studies at 2 and 5 years after hip arthroscopy in patients with various types of FAIS patients have demonstrated favourable PROs, with younger age and shorter symptom duration associated with better outcomes [11, 20, 31, 43]. In our study, patients with mixed FAIS similarly experienced significant improvements in PROs posthip arthroscopy, with the majority achieving PASS and MCID. These results are not only statistically significant but also clinically meaningful.

This study has several limitations. First, all patients were obtained from the single institution, which makes external validation difficult, and the results need to be interpreted with caution. Our models have high internal validity and may be accurate only in patients with mixed FAIS undergoing hip arthroscopy at our centre, as surgical techniques and imaging measurement details such as the alpha angle may have centre‐specific characteristics. Second, the limited number of available predictive factors constrains comprehensive analysis. For instance, the absence of information on patients' daily activity habits hinders understanding their potential impacts and providing targeted recommendations. Thirdly, sample imbalance may overestimate the model's performance, requiring careful interpretation of its classification capabilities. While the SMOTE algorithm aims to balance minority samples, its application may not fully reflect real‐world scenarios.

## CONCLUSION

Three ML prediction models were developed to predict the outcomes of patients with mixed‐type FAIS after hip arthroscopy. Among them, RF performed best in predicting whether PROs reached MCID, demonstrating excellent discriminative ability, calibration and robustness. This indicates that individualised and robust ML prediction models for outcome prediction based on preoperative factors are feasible even with limited amounts of centre‐specific data. Surgeons can use the clinical data of their own centres to establish centre‐specific prediction models, so as to make accurate prediction of postoperative outcomes for their patients.

## AUTHOR CONTRIBUTIONS

Conceptualisation was permormed by Gang Yang, Jiali Kang, Fan Hu and Xin Zhang. Material preparation, data collection and analysis were performed by Yin Pei, Dingge Liu, Zhihua Zhang, Kaiping Liu, Langran Wang, Xi Gong, Haijun Wang, Shuangshuang Deng, Ruijie Liu and Jiali Kang; The first draft of the manuscript was written by Gang Yang, Jiali Kang, Fan Hu, Xin Zhang and all authors commented on previous versions of the manuscript. All authors read and approved the final manuscript.

## CONFLICT OF INTEREST STATEMENT

The authors declare no conflict of interest.

## ETHICS STATEMENT

Approval for the study was granted through the Peking University Third Hospital review board IRB (Number: M2019193).

## Data Availability

The data that support the findings of this study are available from the corresponding author upon reasonable request.
